# Correlates of tobacco cessation counseling among Hispanic physicians in the US: A cross-sectional survey study

**DOI:** 10.1186/1471-2458-8-5

**Published:** 2008-01-08

**Authors:** Francisco G Soto Mas, Héctor G Balcázar, Julia Valderrama Alberola, Chiehwen Ed Hsu

**Affiliations:** 1 College of Education, University of Texas at El Paso, Texas, USA; 2 University of Texas Houston School of Public Health El Paso Regional Campus, Texas, USA; 3 International Health Consultant, Madrid, Spain; 4 UT School of Health Information Sciences, University of Texas Health Science Center at Houston, Texas, USA; 5 University of Maryland School of Public Health, College Park, Maryland. USA

## Abstract

**Background:**

Physician advice is an important motivator for attempting to stop smoking. However, physicians' lack of intervention with smokers has only modestly improved in the last decade. Although the literature includes extensive research in the area of the smoking intervention practices of clinicians, few studies have focused on Hispanic physicians. The purpose of this study was to explore the correlates of tobacco cessation counseling practices among Hispanic physicians in the US.

**Methods:**

Data were collected through a validated survey instrument among a cross-sectional sample of self-reported Hispanic physicians practicing in New Mexico, and who were members of the New Mexico Hispanic Medical Society in the year 2001. Domains of interest included counseling practices, self-efficacy, attitudes/responsibility, and knowledge/skills. Returned surveys were analyzed to obtain frequencies and descriptive statistics for each survey item. Other analyses included: bivariate Pearson's correlation, factorial ANOVAs, and multiple linear regressions.

**Results:**

Respondents (n = 45) reported a low level of compliance with tobacco control guidelines and recommendations. Results indicate that physicians' familiarity with standard cessation protocols has a significant effect on their tobacco-related practices (r = .35, variance shared = 12%). Self-efficacy and gender were both significantly correlated to tobacco related practices (r = .42, variance shared = 17%). A significant correlation was also found between self-efficacy and knowledge/skills (r = .60, variance shared = 36%). Attitudes/responsibility was not significantly correlated with any of the other measures.

**Conclusion:**

More resources should be dedicated to training Hispanic physicians in tobacco intervention. Training may facilitate practice by increasing knowledge, developing skills and, ultimately, enhancing feelings of self-efficacy.

## Background

Despite efforts by federal health agencies, physicians' lack of intervention with smokers has only modestly improved in the last decade [1-3]. Many studies have explored the reasons why physicians do not follow tobacco counseling recommendations. These include physicians' belief that their patients do not want any assistance in quitting smoking [4]; lack of knowledge to provide smoking cessation [5-8]; negative attitudes toward smoking cessation counseling [9,10]; lack of training in smoking-cessation counseling [11]; and lack of familiarity with smoking counseling and treatment guidelines [12]. Another issue that deserves attention is the importance of system-related barriers to tobacco counseling. The US health care system "*has not responded to the frequent calls for action in addressing tobacco use *[13]." Physicians, including Hispanic physicians, continue to complain about lack of time, absence of economic reimbursement, and inappropriate training for effectively providing counseling to smoker patients [14-16].

Although the literature includes extensive research in the area of the smoking intervention practices of clinicians, few studies have explored the tobacco counseling practices of Hispanic physicians. A preliminary study by this research team found that Hispanic physicians, like US physicians in general, did not report the level of intervention recommended by health care agencies [16]. However the literature lacks specific information on the factors that may be associated with tobacco counseling practices among Hispanic physicians. This is an important limitation given that this group constitutes a considerable professional collective: more than 46,000 according to the American Medical  Association [17]. Additionally, the literature supports the need for tobacco-related studies involving Hispanic physicians [16,18]. Not only is it true that tobacco use constitutes a public health priority among Hispanics in the US, where there are more than 6 million adolescent and adult Hispanic smokers [19,20], but Hispanics are also less likely to receive advice to quit [21]. Both the 1992 and the 2000 National Health Interview Survey (NHIS) found that Hispanic smokers were less likely to receive physician advice to quit, in comparison to other racial/ethnic groups [21]. Furthermore, it has been shown that race, culture, and language are important factors among Hispanics when selecting their physicians [22-24]. Should Hispanics prefer physicians who share their same ethnicity, the Hispanic physician may be most suited to attend to the smoking cessation needs of Hispanic smokers [16].

There exists therefore, a need for studies that explore the factors associated with Hispanic physicians' tobacco cessation counseling practices. The purpose of this study was to explore the correlates of tobacco counseling practices among Hispanic physicians. Tobacco use counseling practices were also assessed.

## Method

Research design and methods for this study have been described elsewhere [16] and will only be summarized here. The study was approved by the University of New Mexico Institutional Review Board. Data were collected through a validated survey instrument among a cross-sectional sample of self-reported Hispanic physicians. Participants were asked to sign and return an institutionally approved informed consent that was attached to the survey. Data were collected in 2001, and the study completed in 2002.

### Sample

Participants included self-reported Hispanic physicians practicing in New Mexico (NM) who were members of the NM Hispanic Medical Society (NMHMS). Eighty-one members of the NMHMS qualified for the study.

### Procedures

A packet including a cover letter, the approved informed consent, the survey, and a self-stamped return envelope was mailed to all eligible physicians. After a month, a reminder letter was sent to those who had not returned the survey at that time. A second packet was mailed a few weeks later to 25 physicians, who had not responded to the first request. This packet included a letter offering an incentive to participate.

### Instrument

Data were collected using an instrument developed by the investigators, and which has demonstrated good validity and reliability [16]. Participants were asked to report their ethnicity by checking "Hispanic/Latino" or "Other." The survey also included an assessment of participants' smoking status. In accordance with the literature, the domains of interest for this study included self-efficacy, attitudes/responsibility, and knowledge/skills. Attitudes/responsibility was assessed by asking respondents: a) how they perceive their responsibility for providing counseling to smoking patients, b) how they perceive patients' expectations about physician counseling, and c) whether they perceive their intervention practices as being successful in increasing quitting rates. Physicians were asked to indicate whether they strongly agreed = 4, agreed = 3, disagreed = 2, or strongly disagreed = 1 with each one of the three statements indicated above.

Three items assessed self-efficacy. These included respondents' confidence in being able to: a) get smoking patients to quit their habit, b) get smoking patients to reduce the number of cigarettes smoked daily, and c) reduce patient exposure to secondhand smoke. This outcome-based conception of self-efficacy is supported by both the health and education literature [25-27]. Physicians were asked to indicate their level of confidence on an incremental Likert-type scale that ranged from not confident at all (value = 1) to very confident (value = 4).

Knowledge and skills were assessed through questions related to degree of familiarity with and use of extensively disseminated smoking cessation protocols and theories among US health care providers. These include: the Agency for Healthcare Research and Quality's (AHRQ)*Treating Tobacco Use and Dependence: Clinical Practice Guideline *(which was updated in October 2000 by the U.S. Department of Health and Human Services, Public Health Service) [50]; the National Cancer Institute's (NCI) *4 A's of Smoking Cessation Counseling *(later updated to 5 A's) [51]; the American Lung Association's (ALA) *Freedom From Smoking *[52]; and the American Cancer Society's (ACS) *Fresh Start Family *(no longer available on the ACS website). In addition, participants were asked to indicate their level of familiarity with and use of the Transtheoretical Model (TM) or stages of change model [28]. The scale ranged from very familiar and know how to apply it = 3, familiar but don't really know how to apply it = 2, and not familiar at all = 1.

Additionally, nine items assessed tobacco counseling practices for all patients as well as for smoking patients. These included cigarette smoking, exposure to secondhand smoke, nicotine replacement therapy and other cessation treatments, and behavioral change techniques and programs. Respondents were asked to indicate the percentage of patients with whom they perform each activity in a typical office visit according to five ordinal scales, ranging from "less than 20%" to "more than 80%." The "more than 80%" criteria used as the standard for defining routine practice was based on the *Healthy People 2000 Objectives for the Nation*, which established 75% as the benchmark for "routine" tobacco-related practices.

### Data management and analysis

Returned surveys were analyzed to obtain frequencies and descriptive statistics for each item. Other analyses included: factorial ANOVAs, bivariate Pearson's correlation and multiple linear regressions. All data screening, computation, and analyses were conducted using SPSS 10.0 (SPSS, Chicago, Illinois) for Microsoft Windows. Responses for the four domains of interest (tobacco-related practices, self-efficacy, responsibility/attitudes and knowledge) were measured on an ordinal, incremental scale: the higher the number the more positive the item response. Scores were computed as the sum of the individual items. Four factorial analyses of variance were conducted to investigate significant mean score differences between demographic characteristics and the domains of interest. Bivariate Pearson's r correlations were computed between all measure scores to assess correlation between the domains of interest.

Additionally, to establish potential correlates of counseling practice, a backward multiple linear regression was computed, using attitudes/responsibility, self-efficacy, knowledge/skills, and gender as predictor variables, and tobacco-related practices as the criterion variable.

## Results

### Descriptive statistics

The response rate was 55.5% (n = 45). Ten completed surveys were returned after the second request was mailed out. Characteristics of respondent physicians are included in Table 1. The majority of respondents were male, in the 36–45 years-of-age group, and born in the US. The number of participants who were specialists was slightly higher than that of those who were primary care physicians. None reported being current cigarette smokers at the time of completing the survey, although 26.7% (n = 12) had smoked more than 100 cigarettes in their lifetime.

**Table 1:** Characteristics of Respondents

Item	n (%)
Gender	
Female	12 (26.7)
Male	33 (73.3)
Age group	
20–35	1 (2.2)
36–45	22 (48.9)
46–50	9 (20.0)
50+	13 (28.9)
Place of Birth	
USA	41 (91.1)
Mexico	2 (4.4)
Puerto Rico	2 (4.4)
Professional Category	
Primary care	21 (46.7)
Specialist	24 (53.3)
Years of Practice	
1–3	3 (7.0)
3–6	1 (2.3)
6–10	8 (18.6)
10+	31 (72.1)
Type of Practice	
Private office	23 (53.5)
HMO	2 (4.7)
Hospital	7 (16.3)
Non-hospital based clinic	4 (9.3)
Country of medical training	
USA	42 (95.2)
Mexico	1 (2.3)
Puerto Rico	1 (2.3)
Language spoken at home	
English	41 (91.1)
Spanish	1 (2.2)
Both	12 (6.7)
Language most spoken in practice	
English	35 (77.8)
Spanish	2 (4.4)
Both	8 (17.8)

Tobacco-related practices among respondents, the criterion variable, were previously reported elsewhere [16]: In brief, respondents reported a low level of compliance with tobacco control guidelines and recommendations made by federal health agencies. Fewer than 44% routinely performed the most basic interventions: asking patients about smoking status and advising smoking patients to quit; 24% routinely assisted smoking patients by talking to them about the health risks of smoking, providing education materials or referring them to cessation programs; 4% routinely arranged follow-up visits or phone calls for smoking patients; 36% routinely prescribed cessation medications to smoking patients; only 4% used behavior change techniques or referred smokers to programs that use behavior change approaches to facilitate smoking cessation; and 15% asked patients about exposure to secondhand smoke [16].

The analysis focused on the factors associated with physicians' tobacco counseling practices, including attitudes/responsibility, self-efficacy and knowledge/skills. The majority of participants (90%) felt that it is the responsibility of physicians to provide counseling to patients who smoke. More than 71% agreed that most patients expect them to provide smoking counseling, and 73% agreed that the advice of a physician increases quitting rates. However, participants' responses to self-efficacy-related questions revealed that only 27% were confident about getting patients to reduce daily smoking, 11% were confident about being able to get their smoking patients to quit, and 20% were confident about being able to reduce patients' exposure to secondhand smoke.

Regarding familiarity with smoking cessation protocols and theories, although approximately 40% of respondents were familiar with AHRQ's *Clinical Practice Guideline*, only 7% indicated that they would know how to use it. Sixty-eight percent were not familiar with the *4 A's *or the *Freedom From Smoking*, and 80% were not familiar with the *Fresh Start Family*. Twenty percent were familiar with the stages of change theory but did not know how to apply it, and 78% were not familiar with the theory at all.

### Score distributions

Frequency tables of the score distributions for the measures were examined for out-of-range scores and none were found (see Table 2). Distributions were examined for outlying scores, that is those scores that were three standard deviations above or below the mean for each measure. No outliers were found. All score distributions were within the range of normality as indicated by their values for skew and kurtosis, with the exception of the score distribution for attitudes/responsibility which had a skew value of -1.61 and a kurtosis value of 5.29 (see Table 3). However, examining the actual frequency distribution of attitude scores indicated that the abnormality was not very serious. Examination of the bivariate scatterplots of self-efficacy, attitudes/responsibility, and knowledge/skills scores with tobacco-related practice scores indicated reasonably linear relationships between each of the pairs of measure scores. Means and standard deviations for the four measures by gender were computed (see Table 4).

**Table 2:** Possible and Actual Score Ranges for the Measures (Domains)

		**Possible Score Range**		**Actual Score Range**	
**Measure (Domain)**	**N**	**Max**	**Min**	**Max**	**Min**
Tobacco-Related Practices (8 items)	45	40	8	32	8
Self-Efficacy (3 items)	45	12	3	9	3
Attitudes/Responsibility (3 items)	45	12	3	12	3
Knowledge/Skills (5 items)	45	15	5	12	5

**Table 3:** Descriptive Statistics for Measures

**Measure (Domain)**	**N**	**Mean**	**Std. Dev.**	**Std. Error**	**Skew**	**Kurtosis**
Tobacco-Related Practices	45	18.75	6.79	1.01	0.20	-0.65
Self-Efficacy	45	6.02	1.63	0.24	-0.27	-0.33
Attitudes/Responsibility	45	8.86	1.50	0.22	-1.61	5.29
Knowledge/Skills	45	6.70	1.95	0.29	1.00	-0.00

**Table 4:** Means and Standard Deviations of Measure Scores by Gender

**Measures (Domains)**	**Gender**					
	**Male (n = 33)**		**Female (n = 12)**		**Total (n = 45)**	
	**Mean**	**Std. Deviation**	**Mean**	**Std. Deviation**	**Mean**	**Std. Deviation**
Tobacco-Related Practices	20.20	6.50	14.75	6.14	18.75	6.79
Self-efficacy	6.15	1.73	5.68	1.30	6.02	1.63
Attitudes/Responsibility	8.64	1.54	9.46	1.24	8.86	1.50
Knowledge/Skills	6.93	2.06	6.08	1.51	6.70	1.95

### Correlations

Significant correlations were found between tobacco-related practices and self-efficacy scores (r = .42, variance shared = 17%), between tobacco-related practices and knowledge/skills (r = .35, variance shared = 12%), and between self-efficacy and knowledge/skills (r = .60, variance shared = 36%). [Note: the score variance shared between two measures is computed by squaring the Pearson's r correlation value.] Interestingly, attitudes/responsibility was not significantly correlated with any of the other measures. In addition, age group was not significantly related to any of the four measures (see Table 5).

**Table 5:** Pearson's r Correlations Between all Measures

	**Tobacco-Related Practices**	**Self-Efficacy**	**Attitudes/Resp.**
Self-Efficacy	.415**		
Attitudes/Resp.	.102	.142	
Knowledge/Skills	.351*	.602**	.044

** Correlation is significant at the 0.01 level (2-tailed).

* Correlation is significant at the 0.05 level (2-tailed).

### Factorial ANOVAs

Four factorial analyses of variance were conducted to investigate if there were significant mean score differences on any of the four measures by gender (male/female), by type of physician (primary care or not), or by the interaction of gender and type of physician. Results indicated a main effect of gender on tobacco-related practice, with male physicians scoring significantly more positively than female physicians. Male physicians (M = 20.20, SD = 6.50) scored nearly 30% higher than female physicians (M = 14.75, SD = 6.14) (see Table 4). There was no significant main effect of type of physician, nor was there an interaction effect (gender by type of physician), on tobacco-related practices (see Table 6). In the three other factorial analyses, no interaction effects or main effects of gender or physician type on mean scores of attitudes/responsibility, knowledge/skills, or self-efficacy measures were found.

**Table 6:** Factorial ANOVA Results Indicating Main and Interaction Effects of Gender and Type of Physician on Tobacco-Related Practices

**Source of Variance**	**Sum of Squares**	**df**	**Mean Square**	**F**	**p**
Gender	287.89	1	287.89	6.87	.01*
Type of Physician	37.38	1	37.38	.89	.35
Gender by Type of Physician	32.24	1	32.24	.77	.39
Error	1717.12	41	41.88		

R Squared = .152

### Regressions

Results indicated that 29% of the variance in tobacco-related practice scores could be explained by using the four independent variables. Gender (male) was the single independent variable that was a significant predictor of, or explained a significant amount of variance in, tobacco-related practices (Model 1). When gender and attitudes/responsibility and self-efficacy were used as independent variables (dropping knowledge/skills) (Model 2), only 1% less variance was explained. Self-efficacy and gender were both significantly correlated to tobacco related practices, but attitudes/responsibility was not. Finally, when attitudes/responsibility was also dropped as an independent variable (Model 3), gender and self-efficacy were left as significant correlates, together explaining 27% of the variance in tobacco-related practices scores (see Table 7). Further analyses found that gender uniquely accounted for approximately 10% of the variance in tobacco-related practices scores, and self-efficacy uniquely accounted for approximately 14%, with approximately 3% of the variance in tobacco-related practices explained by the overlap of gender and self-efficacy.

**Table 7:** Multiple Linear Regression Coefficients for Model 3

	**Unstandardized Coefficients**		**Standardized Coefficients**		
	**b**	**Std. Error**	**Beta**	**t**	**p**
Gender	-4.71	2.02	-.31	-2.33	.03*
Self-Efficacy	1.56	0.56	.37	2.81	.01**

Dependent Variable: Tobacco-Related Practice

**p ≤ 0.01.

*p ≤ 0.05.

## Discussion

To the knowledge of these investigators, this is the first study assessing the factors that may be associated with tobacco counseling practices among Hispanic physicians in the US. Despite the exploratory nature and limitations of the study, results may assist in the developing of effective approaches for training Hispanic physicians in tobacco counseling. Given the important role physicians play in smoking cessation [13,29], better trained Hispanic physicians may positively impact tobacco use among Hispanic patients. Additionally, this study provided preliminary data which support the need for future research related to Hispanic physicians and tobacco control.

The majority (73%) of respondents recognized that the advice of physicians increases quitting rates. These results are consistent with other published papers reporting on high perceived responsibility to educate patients who smoke [30]. Although some authors have reported an association between perceived professional responsibility [31] and attitudes towards smoking cessation counseling [9], the present study did not find a significant correlation between responsibility/attitudes and tobacco-related practices. Furthermore, this variable was not significantly correlated with any of the other two domains: knowledge/skills and self-efficacy.

Regarding self-efficacy, respondents had low confidence in convincing smoker patients to quit or lower their exposure to secondhand smoke. This study found a significant correlation between tobacco practices and self-efficacy, which is consistent with the literature. Physicians who report a feeling of self-efficacy in the area of tobacco use and prevention are in fact more likely to intervene with patients [5,32,33]. Self-efficacy is defined as the confidence a person has that he/she can perform a particular activity, and overcome challenging situations to perform that activity [34,35].

Results indicate that physicians' familiarity with standard cessation protocols has a significant effect on their tobacco-related practices. These results are consistent with other studies that have reported significant correlations between practice and knowledge, as well as skills and training [3,33,36-40]. Although many tobacco-related studies have found significant correlations between knowledge and practice, it is generally assumed that knowledge is not independently predictive of behavior. Many authors have argued that clinicians with greater knowledge have probably received more training and are more committed and prepared to intervene [5]. In this study, knowledge/skills was also significantly correlated with self-efficacy, which may explain why participants with greater knowledge also reported better performance.

As regards the TM, none of the respondents were familiar enough with the TM to use it. These results are consistent with the literature, which has reported on the low use by physicians of evidence-based approaches to smoking cessation [15,39,41], and on the ineffectiveness of professional/clinical practice guidelines in changing physicians' practices [38,42,43].

Finally, the results of the multiple linear regressions revealed that the two single independent variables that best correlated with practice were self-efficacy and gender. Other studies among health providers have found no gender differences in tobacco performance [5], and indicate that gender does not predict self-reported smoking counseling behavior [38]. A recent study found that female physicians were more active in counseling patients on smoking and other preventive behaviors [44]. In contrast, this study found better practice among participant male physicians. These differences may be due to the cultural background of our sample, consisting of physicians of Hispanic heritage: this issue warrants further research. Note that according to the US Office of Personnel Management, "Hispanic" is used to refer to "a person of Mexican, Puerto Rican, Cuban, Central or South American or Spanish culture or origin, regardless of race [45]." This study asked participants to report their Ethnicity by checking "Hispanic/Latino" or "Other."

## Limitations

First, the sample size was small, and limited to providers practicing in New Mexico. Therefore generalization of findings must be made cautiously. We did estimate the representation of respondents in relation to the overall number of physicians in NM and nationally at the time of the study. Nationally, Hispanics represented 3% of the physicians registered with the American Medical Association [46]. According to the NM Board of Medical Examiners [47], the number of practicing physicians was 3,600, of which approximately 10% were Hispanic. Since 45 physicians responded to the survey, approximately 13% of the Hispanic physicians practicing in NM participated in the study. Second, virtually all participants were born in the US, which may also constitute a limitation to the generalization of results. Approximately 25% of the physicians working in the US are foreign born [48], and many were educated in Latin America and Spain. These physicians may have different perceptions and attitudes regarding tobacco counseling. Third, survey data were self-reported. Although the survey instrument demonstrated good validity and reliability, providers' self-reported practices may be less valid than data obtained from other sources [49].

## Conclusion

More resources should be dedicated to training Hispanic physicians in tobacco intervention. Training may facilitate practice by increasing knowledge, developing skills and, ultimately, enhancing feelings of self-efficacy. In addition to training, counseling practices must be supported by system-based changes. More studies are needed to further explore whether: a) female Hispanic physicians are in need of increased training for tobacco counseling, b) perceived responsibility for intervening with patients who smoke improves practice among Hispanic physicians, and c) self-efficacy levels compare to that of physicians of other racial/ethnic groups.

## Abbreviations

ACS: American Cancer Society

AHRQ: Agency for Healthcare Research and Quality

ALA: American Lung Association

NCI: National Cancer Institute

NHIS: National Health Interview Survey

NM: New Mexico

NMHMS: New Mexico Hispanic Medical Society

TM: Transtheoretical Model

US: United States

## Competing interests

The author(s) declare that they have no competing interests.

## Authors' contributions

FGSM designed the study, conducted data collection and analysis, and wrote the first draft of the manuscript. HB contributed to data analysis, and technical and editorial review. JVA contributed to study design and editorial review. CEH contributed to technical, including statistical and editorial review. All authors read and approved the final manuscript.

## Pre-publication history

The pre-publication history for this paper can be accessed here:


